# Adnexal carcinomas of the head and neck

**DOI:** 10.4103/0970-0358.44950

**Published:** 2008

**Authors:** Jorge O. Güerrissi, Juan Pablo Quiroga

**Affiliations:** Argerich Hospital, Buenos Aires, Argentina

**Keywords:** Adnexal carcinomas, adnexal tumors, skin cancer

## Abstract

Adnexal carcinomas of the skin are rare and they derive from structures such as sweat glands, sebaceous glands, and hair follicles. Adnexal tumors represent 1–2% of skin cancers. Between 1998 and 2004, eight patients with malignant adnexal tumors of the head and neck were treated in the Plastic Surgery Service in Argerich Hospital in Buenos Aires, Argentina. Four (50%) of them had malignant cylindromas, two (25%) had sebaceous carcinoma, and the other two (25%) syringoid eccrine carcinoma. Tumor resection and local flaps were made in all cases. In one case, a radical neck dissection with superficial parotidectomy was performed to treat the metastatic cervical nodes. Local recurrence observed in two cases (25%) was associated with distant metastasis and death of the patients. In other six cases, the survival rate was 75% after five years.

## INTRODUCTION

Melanoma skin cancers account for 4–7% of all skin cancers, the non melanoma tumours for 93–96%. Both basal cell and squamous cell carcinomas represent > 80% of non melanoma skin cancers whereas benign and malign adnexal tumours represent only 1–2%, including mesenchymal, fatty, and vascular tumours. Adnexal carcinomas of the skin derive from structures that have a common origin such as the apocrine and eccrine sweat glands, sebaceous glands, and hair follicles [Table 1]. Malignant adnexal tumours are frequently located in the head and neck region but may appear on the fingers and toes, the trunk as well as the extremities.[1–4]

**Table 1:** Adnexal carcinomas of the skin

ADNEXAL CARCINOMAS		

*Sebaceous tumors*	*Hair follicle tumors*	*Sweat gland tumors*
1. Sebaceous	1. Merkel Cell Carcinoma	Low grade
Carcinoma	2. Pilomatrixoma and pilomatrix	1. Eccrine epithelioma
	Carcinoma	2. Adenoidocystic Carcinoma
		3. Mucinous Carcinoma
		4. Extramammary Paget
		Intermediate grade
		1. Porocarcinoma
		2. Digital Papillary Adenocarcinoma
		High grade
		1. Apocrine Adenocarcinoma
		2. Eccrine duct Carcinoma
		3. Spiroadenocarcinoma
		4. Malignant Cylindroma

*RND+SP: Radical Neck Dissection and Superficial Parotidectomy

All ages may be affected and there is no easily identifiable risk group.

Most adnexal carcinomas of the skin are highly malignant and difficult to diagnose clinically or even histologically.

The objective of this presentation is to attract the attention of plastic surgeons because a reliable cure rate is associated with prompt recognition and aggressive treatment.

## MATERIAL AND METHODS

Between 1998 and 2004, eight patients with adnexal carcinomas of the skin in the head and neck region were admitted to the Service of Plastic Surgery in the Argerich Hospital. Four (50%) of them were diagnosed with malignant cylindromas; two (25%) with sebaceous carcinoma, and the remaining two (25%) with syringoid eccrine carcinoma. The patients´ ages ranged from 26 to 68 years and the follow-up period was for five years.

Local recurrence was observed in two cases (25%) with malignant cylindroma and syringomoid eccrine carcinoma.

The treatment of choice was a wide excision of the tumour (> 1.5 cm) and local flaps for reconstruction in seven cases and a split-thickness skin graft in the other case.

A radical neck dissection including superficial parotidectomy was done in one of the two cases of sebaceous carcinoma when metastatic cervical nodes were detected.

Six patients (75%) were followed up for at least five years; two others (25%) died of local recurrence and distant metastasis within a mean follow-up period of 36 months [Table 2].

**Table 2:** Surgical treatment and Follow-up

*Case*	*Age/ Sex*	*Site*	*Histology*	*Treatment*	*Recurrence*	*Survival*
1	52/M	Scalp	Malignant Cylindroma	Resection + Flap	Yes +Metastasis	1 year
2	34/F	Cheek	Malignant Cylindroma	Resection + Flap	No	5 years
3	26/F	Scalp	Malignant Cylindroma	Resection + Flap	No	5 years
4	57/M	Scalp	Malignant Cylindroma	Resection + Flap	No	5 years
5	68/F	Upper Lid	Sebaceous Cylindroma	Resection + Flap	No	5 years
				RND+SP *
6	49/F	Cheek	Sebaceous Carcinoma	Resection + Flap	No	5 years
7	47/F	Scalp	Syringoma	Resection + Flap	Yes + Metastasis	3 years
8	50/M	Nose	Syringoma	Resection + Skin graft	No	5 years

In these two patients, chemotherapy and radiotherapy were used with very limited success.

Major complications were not observed and only two cases presented with infection of the incision.

### Malignant cylindroma

**Figure 1:**
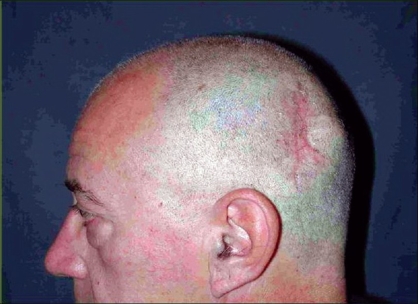
Cylindroma of the scalp

**Figure 2:**
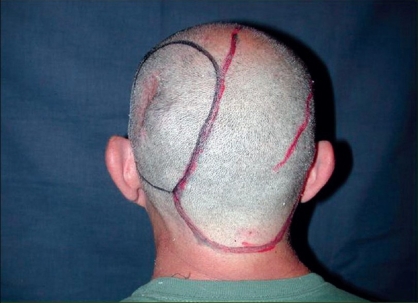
Area of resection and drawing flaps of the scalp

**Figure 3:**
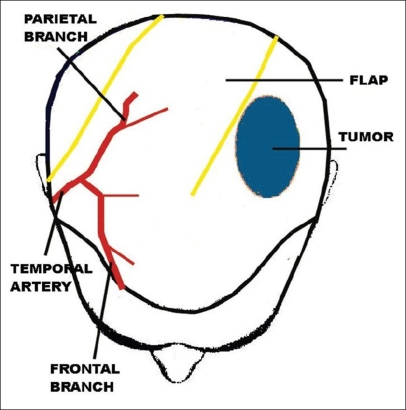
Schematic drawing of the scalp flap

**Figure 4:**
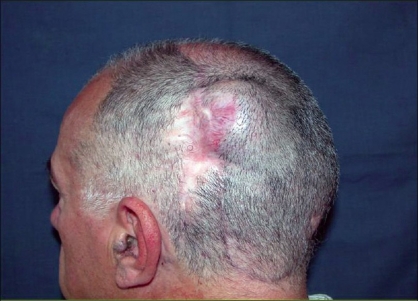
Aggressive local recurrence

**Case 1:** A 52 year-old man presented a recurrent malignant cylindroma on his scalp [Figure 1] Excision of the tumour and a pedicled scalp flap based on the superficial temporal artery was performed [Figures 2–3]. Histological findings showed multiple compact epithelial lobes with central cells with wide nuclei and peripheral cells with small and dark nuclei. Approximately after two years, the patient presented the first recurrence at the level of the upper insertion of the trapezius muscle and a new surgical excision was carried out. After five months, a new and very aggressive recurrence was exhibited that involved the brain and the meninges as well as distant metastasis [Figure 4]. Adjuvant radiotherapy was unsuccessfully applied on the local recurrent lesion but he died nine months after the last surgery.

### Sebaceous carcinoma

**Figure 5:**
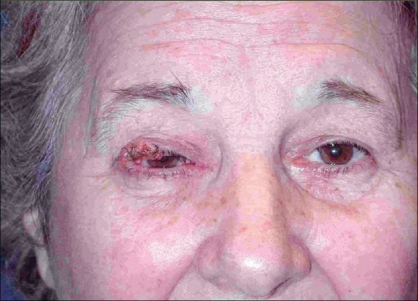
Sebaceous carcinoma on the superior right eyelid superior

**Figure 6:**
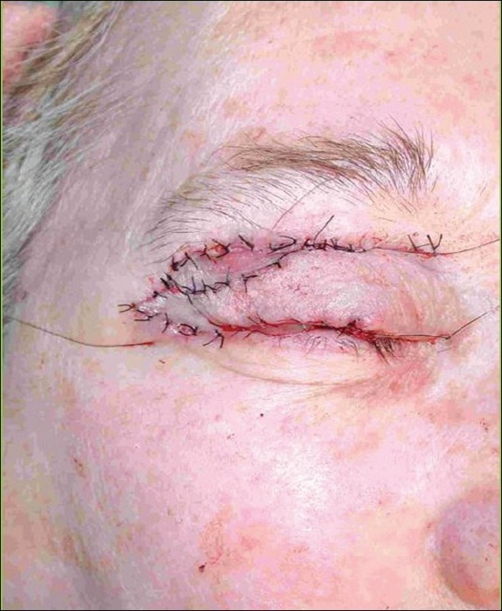
Eyelid reconstruction by means of an orbicularioculis musculocutaneous flap

**Figure 7:**
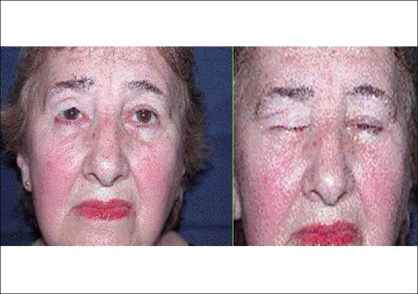
Postsurgical result

**Case 2:** A 68 year-old woman presented with a tumour at the border of the right superior eyelid. Biopsy results indicated a carcinoma of the sebaceous cells [Figure 5]. Complete tumor resection was carried out and eyelid reconstruction was performed with graft of the palate mucosa and an ipsilateral orbicularis oculi musculocutaneous flap [Figure 6]. Metastatic cervical nodes were detected after two months, and a radical neck dissection was done with superficial parotidectomy. Excellent aesthetic and functional results were obtained after five years [Figure 7].

### Syringoid eccrine carcinoma

**Figure 8:**
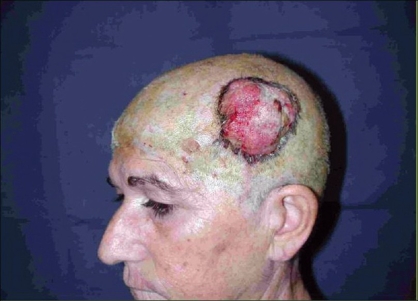
Syringoid eccrine carcinoma of the scalp

**Figure 9:**
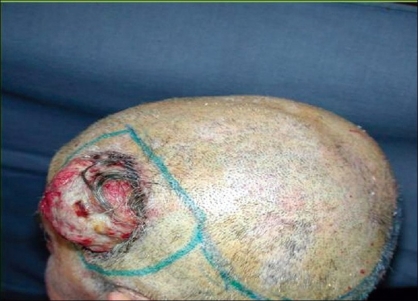
Resection of the tumor and a designed scalp flap

**Figure 10:**
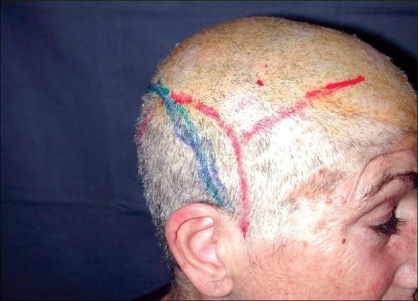
Irrigation of the scalp flap

**Figure 11:**
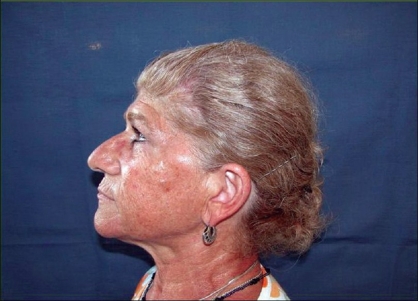
Postsurgical result after two years

**Case 3:** A 47 year-old woman presented with a tumour of the scalp. Biopsy results indicated a syringoid eccrine carcinoma [Figure 8]. A wide tumour excision was planned [Figure 9] and a scalp flap was designed to cover the defect based on the superficial temporary artery [Figure 10]. Excellent aesthetic and functional results were obtained and recurrence was not detected after five years [Figure 11].

## DISCUSSION

The adnexal carcinomas of the skin are not frequent and they derive from the foetal epidermis.[16] Some have a predilection for certain specific locations such as syringomas of the cheek and nose; cylindromas of the scalp and face; sebaceous carcinoma in the eyelids, etc.

Clinical diagnosis of most adnexal skin carcinomas is difficult not only between them but also with adnexal benign tumours.

Factors suggesting malignancy include poor demarcation, tumour necrosis, ulcers, border, tumour infiltration, etc.

Malignant cylindroma is a rare tumour derived from the eccrine sweat glands.[5] Its origin was formerly considered to be the apocrine sweat gland but is now thought to be due to a mixed aetiology. Only 14 cases are known in literature and they have all presented with aggressive local expansion, perforation of the skull, and metastasis in the cervical nodes.[6–8,10–12] They appear frequently in women and young adults as nodules 1.2–6 cm in diameter; pink in colour and of firm consistency. They may appear in two forms: Solitary (74%), which is the most frequent form seen usually in the face, and multiple (26%) which is of dominant autonomic inheritance, the gene of the familiar cylindromatosis being located in the chromosome 16q12-q13[13] Histology is characterized by the presence of multiple compact epithelial lobes. Two types of cells are described: the central cells with clear nuclei; and the peripheral ones with small and dark nuclei.[14] Differential diagnoses are necessary with other pathologies such as trichoepitheliomas, epitheliomas to basal cell nodules as also metastatic tumours, and trichofolliculomas.

Carcinoma of the sebaceous cells is a very rare tumour. It occurs in women between 60 and 70 years of age and is preferentially located on the eyelids. This carcinoma may appear as a solitary, yellowish, and painless nodule. Histology shows very cellular bands or basophilic masses extended from the dermis to the subcutaneous weave.[15]

Metastasis takes place first in the regional lymphatic nodes and then, in the viscera, these carcinomas can also invade the facial bones.

A syringomoid eccrine carcinoma is also know as a microcystic adnexal carcinoma, malignant syringoma, or a sclerosing carcinoma of the eccrine sweat glands. It is pronounced in aged patients and is usually located on the scalp, face, trunk, and extremities. It is a solitary tumour presenting as a slow-growing nodule or plaque. It can appear with alopecia and the lesion may secrete fluid. The following characteristics are observed in the histological analysis: cellular atypia, nuclear hyperchromatism, deep invasion, and many tubulocystic proliferations at the level of the dermis.[15]

In all our cases of adnexal skin carcinomas of the head and neck region, surgical excision was the treatment of choice, the excision and reconstruction being conducted simultaneously by the same team.

In previous reports, adnexal carcinomas have been considered to be radioresistant although new techniques of radiotherapy have been revaluated as part of primary and adjuvant treatments.[17,18]

Radical neck dissection must be done when metastatic cervical nodes are detected.

## CONCLUSION

- Adnexal skin carcinomas are both very rare and infrequent compared to other nonmelanoma tumors (basal cells and squamous carcinomas).
- The head and the neck region is the favourite site of presentation.
- The treatment of election is a complete surgical resection of the tumor.
- When regional lymphatic metastatic nodes are present, radical neck dissection is the treatment of election.
- Revaluation of adjuvant radiation therapy is presently being considered.
- When the treatment is oncologically sufficient, the result is a safe and long survival.
